# Death of Prof. Worcester

**Published:** 1847-06

**Authors:** 


DEATH OF PROF. WORCESTER.

We regret to learn the death of Dr. Noah Worcester, of Cincinnati, which took place in that city on the 4th of April. Dr. Worcester enjoyed an enviable reputation as an accomplished and skilful physician. He was formerly a professor in the Medical College of Ohio, and at the time of his death held a chair in the Cleveland Medical School, in the same State. He was a native of New Hampshire, and removed to Cincinnati in the fall of 1838. We copy the following paragraphs from a just and very beautiful notice of him in the Cincinnati Gazette:

"Dr. Worcester was no ordinary man. During the nine years of his residence here, he won for himself a reputation of the highest character as a devoted and skilful physician, and a man of enviable attainments in the science of medicine. At the time of his death, his practice was one of the largest in the city; and though cut off thus early, he had gained an eminence not oftentimes attained even at the close of a long career of laborious professional service. He was an indefatigable student as well as a laborious practitioner, and was one of the few who, in the midst of a life of harassing anxieties and unceasing labor, still retain and cultivate with delight an early love for classical studies.

"But it is not merely as an eminent physician and a man of cultivated tastes that his memory will be cherished. There are those to whom he ministered in affliction—with whom he was brought into the nearest and most intimate relations by the duties of his profession. It is upon these that his death falls most heavily; as a bereavement of doubly afflicting power; for it was among these—at the couch of the sufferer, at the family fireside, and in the unrestrained intercourse of private life—that the inestimable worth of the man and sterling virtues of his character were fully developed."

Dr. Worcester published a work on cutaneous diseases, and was also an occasional contributor to different medical journals.

---

